# RGB Color Space-Enhanced Training Data Generation for Cucumber Classification

**DOI:** 10.3390/jimaging11040120

**Published:** 2025-04-17

**Authors:** Hotaka Hoshino, Takuya Shindo, Takefumi Hiraguri, Nobuhiko Itoh

**Affiliations:** Nippon Institute of Technology, 4–1 Gakuendai, Miyashiro, Saitama 345-8501, Japan; 2238013@stu.nit.ac.jp (H.H.); t-shindo@nit.ac.jp (T.S.); hira@nit.ac.jp (T.H.)

**Keywords:** smart agriculture, IoT, machine learning, cucumber

## Abstract

Cucumber farmers classify harvested cucumbers based on specific criteria before they are introduced to the market. During peak harvesting periods, farmers must process a large volume of cucumbers; however, the classification task requires specialized knowledge and experience. This expertise-dependent process poses a significant challenge, as it prevents untrained individuals, including hired workers, from effectively assisting in classification, thereby necessitating that farmers perform the task themselves. To address this issue, this study aims to develop a classification system that enables individuals, regardless of their level of expertise, to accurately classify cucumbers. The proposed system employs a convolutional neural network (CNN) to process cucumber images and generate classification results. The CNN used in this study consists of a total of 11 layers: 2 convolution layers, 2 pooling layers, 3 dense layers, and 4 dropout layers. To facilitate the widespread adoption of this system, improving classification accuracy is imperative. In this paper, we propose a method for embedding information related to cucumber length, bend, and thickness into the background space of cucumber images when creating training data. Specifically, this method encodes these attributes into the RGB color space, allowing the background color to vary based on the cucumber’s length, bend, and thickness. The effectiveness of the proposed method is validated through an evaluation of multi-class classification metrics, including accuracy, recall, precision, and F-measure, using cucumbers classified based on the criteria established by an actual agricultural cooperative. The experimental results demonstrate that the proposed method improves these evaluation metrics, thereby enhancing the overall performance of the system.Specifically, the proposed method achieved 79.1% accuracy, while the method without RGB color space achieved 70.1% accuracy. This indicates that the proposed method achieves 1.1 times better performance than the conventional method.

## 1. Introduction

In response to the aging agricultural workforce and the declining number of workers, attention has shifted to smart agriculture, where robots and Internet of Things (IoT) devices perform agricultural tasks based on sensing information, reducing reliance on human labor [1]. Research in smart agriculture includes strawberry [2] and tomato cultivation [3], with additional studies focused on mini-tomato cultivation [4] and cucumber cultivation. Among these, the authors concentrate on research aimed at replacing farmers’ visual-based judgment tasks. For example, in apple cultivation, there is a need to classify specific types of apples automatically during sorting. A shallow convolutional neural network (CNN) has been proposed for this purpose, achieving approximately 92% classification accuracy for six apple types using a test set [5,6]. Other related research includes disease detection on tomato and cucumber leaves [7,8], okra classification systems [9,10], an automatic carrot classification system [11], a CNN-based chili classification system [12], as well as classification systems for shallots [13] and root-trimmed garlic [14].

Farmer tasks can broadly be divided into preparation, cultivation, harvesting, classification, packing, and shipping, repeated in sequence. The grade of cucumbers, which determines shipment readiness, is based on factors such as length, bend, and thickness, with grading criteria varying by region. Currently, the classification process is performed visually by agricultural workers, who also pack cucumbers into boxes based on classification results. During busy seasons, the need to classify a large number of cucumbers requires substantial time, reducing time available for profit-driven tasks like cultivation and preparation. To reduce the time farmers spend on sorting, hiring specialized personnel for classification could be considered. However, effective sorting requires significant expertise regarding cucumber grades, making it challenging to hire workers who can immediately perform this role. To address this, a classification system using CNNs is under consideration to enable anyone to grade cucumbers easily. However, creating training data for constructing classification models specific to production areas remains a labor-intensive task, posing a significant burden on agricultural workers. Existing research on cucumbers includes the development of a neural network-based automatic inspection system [15], a machine vision-based quality grader [16], and a classifier for desirable (cylindrical) versus undesirable (curved and conical) shapes using image processing and artificial neural networks [17]. Reference [15] proposes a system that measures the geometric characteristics (such as length and shape) of cucumbers in real time while they are moving on a conveyor belt. Reference [16] proposes a novel CNN called MassNet, which is designed to predict the mass (weight) of cucumbers. Reference [17] introduces new shape features to classify cucumber shapes into two classes: “desirable shapes” (cylindrical) and “undesirable shapes” (curved or conical).

In recent years, there have been remarkable advancements in object detection technology using images and videos, and You Only Look Once (YOLO) [18,19] has been proposed as one such object detection engine. YOLO has relatively low computational load, high real-time performance, and is being considered for use in various fields. While YOLO can classify objects into categories such as cars, bicycles, apples, and cucumbers, the cucumber grading classification targeted in this paper cannot be achieved solely by applying the existing YOLO model. However, it is conceivable to develop a classification system based on YOLO by utilizing images processed in our proposed RGB color space. Therefore, rather than being a competing method against YOLO, our proposed approach can be considered a complementary technique that can coexist with it.

In this paper, we propose a method for generating training data images using the RGB color space [20] to achieve more accurate classification and a semi-automatic system for generating training data and learning models. Through evaluation, we validate the system’s accuracy using grading information based on existing agricultural cooperatives and demonstrate the effectiveness of the proposed method.

## 2. Methods

**Figure 1:**
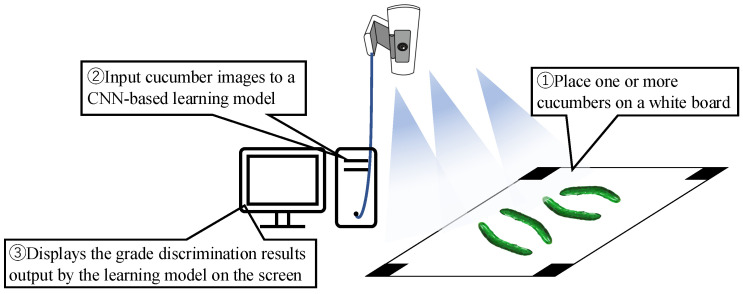
Overview of the cucumber classification system based on image recognition.

Figure 1 shows an overview of the cucumber classification system based on image recognition. After starting the discriminator program, the discriminator places one or more cucumbers on the white board. Markers are placed at the four corners of the white board to accurately measure the size of cucumbers, even when the distance between the camera and white board is not constant. When a cucumber is placed inside the markers on the white board, the cucumber image is input to a learning model constructed using a CNN. The learning model outputs a grade discrimination result.Using this system, no specialized know-how is required for grade identification, enabling anyone to easily identify grades. This reduces the workload of cucumber agricultural workers by allowing the hiring of identification workers. Python 2.8, Tensorflow 2.8.2 [21,22], and OpenCV 4.0.1 [23] were used to create the models.

### 2.1. Generation of Training Data

Generating a learning model requires training data. In the first proposed method, training data consists of an image created by pasting a cut-out image of a cucumber onto a 100 × 340 black background image, numerical information on the height, width, and area extracted from the cucumber-only image, and the correct labels associated with the grade. This method is referred to as without RGB color space. The reason for pasting the image onto the background image is to prevent the size of the cucumber from becoming uniformly scaled across grades during image resizing, which could eliminate critical differences. Image processing is performed using OpenCV, an image processing library.

**Figure 2:**
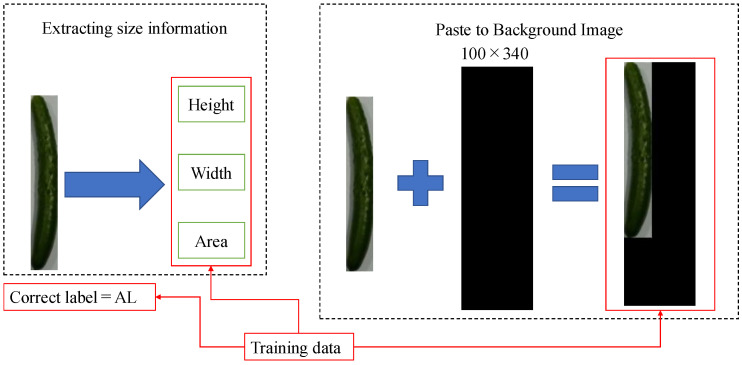
Flow diagram of the method for generating training data images in the method without RGB color space.

Figure 2 shows the flow for generating training data images in the without RGB color space method. The extracted image is resized based on the distance between the markers and the cucumber’s dimensions. Initially, only the cucumber is cropped from the entire screen. Information on the cucumber’s height, width, and area is calculated from the size of the cropped image. The resized image is then pasted onto a uniform black background of 100 × 340 to preserve the cucumber’s size information. Finally, the cucumber image is labeled with the correct grade. The three pieces of information—images, size data, and grade labels—obtained by this method are used as training data to create a learning model.

### 2.2. Generation of the Learning Model

**Figure 3:**
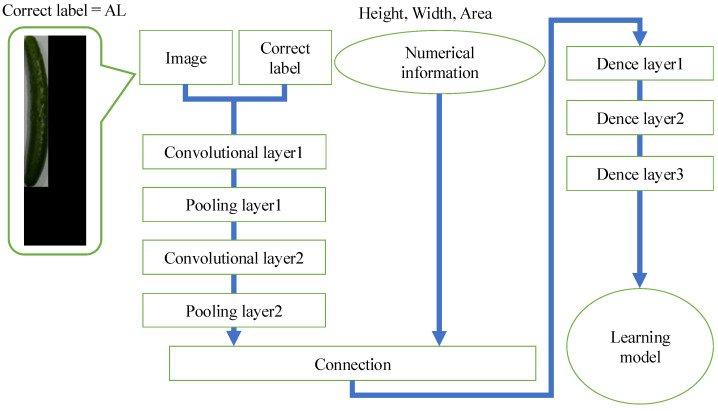
Flow of learning model creation in the method without RGB color space.

Figure 3 shows the flow of learning model creation in the method without RGB color space. The learning model is created using training data (height, width, area, image, and correct answer labels) generated by the method described in Section 2.1. First, the input image is resized to 72 × 24. Next, the resized input images and their associated correct labels are fed into the feature extraction layer, which consists of four layers: two convolution layers and two pooling layers. The extracted features are then combined with the numerical information (height, width, and area) obtained from the cucumber-only image. Finally, the learning model is generated by inputting this concatenated information into a layer comprising three dense layers.

Table 1 shows the Neural Network Parameters. The convolution layer consists of two layers with 8 and 16 filters, respectively, both using a stride of [1, 1]. Rectified linear unit (ReLU) [24] is used as the activation function, and Batch Normalization is applied for regularization. Both pooling layers use a filter size of 2 × 2 and apply max pooling. The dense layer contains three layers, with the first, second, and third layers having 64, 32, and 7 units, respectively. Other parameters are set as follows: the batch size is 100, the maximum number of steps is 10,000, the learning rate is 0.001, the optimizer is Adam, and the loss function is cross-entropy.

### 2.3. Classification

**Figure 4:**
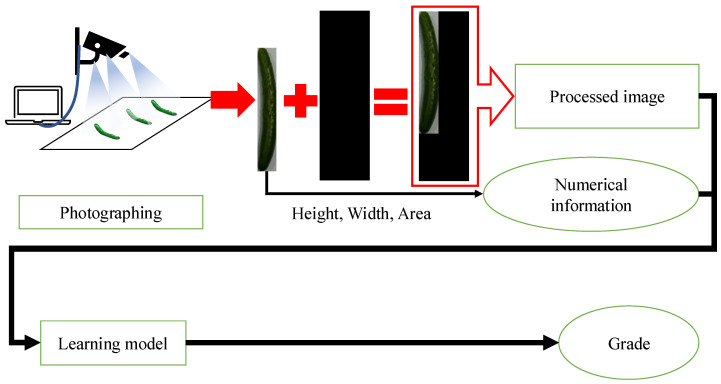
Classification flow in the method without RGB color space.

Figure 4 shows the classification flow in the method without RGB color space. A cucumber placed on a white board is photographed using a webcam connected to a PC. The cucumber is cut out from the captured video to create an image. The cut-out cucumber image is pasted onto a 100 x 340 black background to generate a processed image for use in the judgment process. Next, the height, width, and area information are extracted as numerical values from the cucumber-only image. The processed image and the three extracted size values are input into the learning model created using the method described in Section 2.2. The model then classifies the cucumber and determines its grade. Finally, the grade information is displayed on a PC monitor.

### 2.4. Proposed System

**Figure 5:**
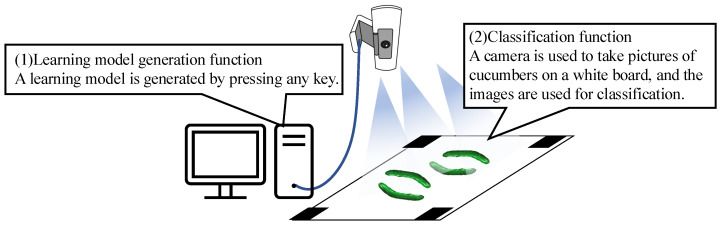
Overview of the proposed system.

Figure 5 presents an overview of the proposed system. The proposed system includes a graphical user interface (GUI) for automatic learning-model generation and a method for generating training data using RGB color space, described in Section 2.5 and Section 2.6, respectively. In this paper, the method for generating training data using RGB color space is referred to as the “method with RGB color space”.

**Figure 6:**
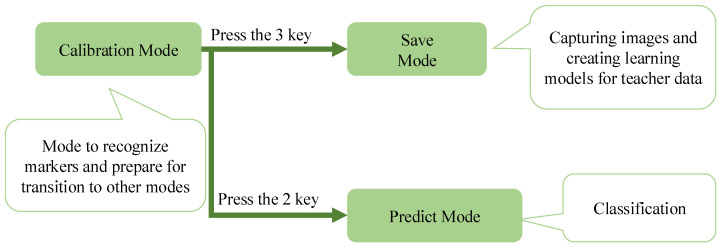
List of modes and mode switches methods for the new device.

Figure 6 shows a list of modes and mode switches methods for the new device. Calibration Mode is activated first when the system starts. In this mode, the four corner markers are recognized, and the mode can be shifted by pressing a specified key. The “2” key is pressed to enter the classification mode, which uses the saved learning model for grading cucumbers. Pressing the “3” key switches to the save mode, where the system described in Section 3.1 is used to capture images for training data and create a learning model.

### 2.5. GUI for Automatic Learning Model Generation

To generate a learning model, training data is required, linking cucumber images with grading information. Creating training data involves photographing cucumbers, cutting out their images, pasting them onto a background, assigning correct labels, and inputting this information into the CNN. However, generating a large quantity of training data, which is necessary for highly accurate classification, poses a significant burden on farmers. To address this challenge, we propose a GUI system that simplifies the entire process, from training data generation to learning model construction.

**Figure 7:**
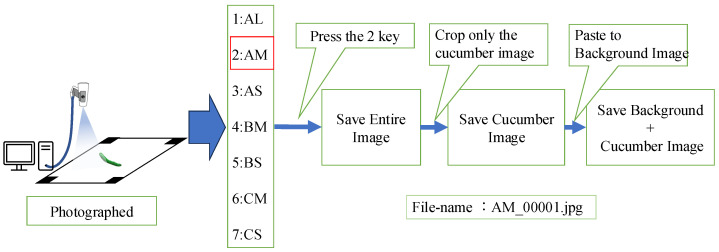
GUI system for automatic learning model generation.

Figure 7 illustrates the GUI system for automatic learning model generation. The GUI semi-automates several tasks: capturing cucumber images, processing them into a trainable format, and assigning the correct grade labels. In this system, cucumber grade information is mapped to a numeric keypad. After placing a cucumber on the white board, the user presses the key corresponding to the cucumber’s grade. When the key is pressed, the system clips only the cucumber from the captured image. The cut-out cucumber image is then pasted onto a 100 × 340 black background. The grade information is embedded at the start of the file name, and both the cut-out image and the pasted image are saved. A sequence number is appended to the file name to prevent duplication. These images are then processed to improve classification accuracy, as detailed in Section 2.6. After the training data is prepared, the system automatically generates and saves a learning model using the stored training data when the Enter key is pressed. The method for learning model generation is described in detail in Section 2.7.

### 2.6. Generation of Training Data Using RGB Color Space

**Figure 8:**
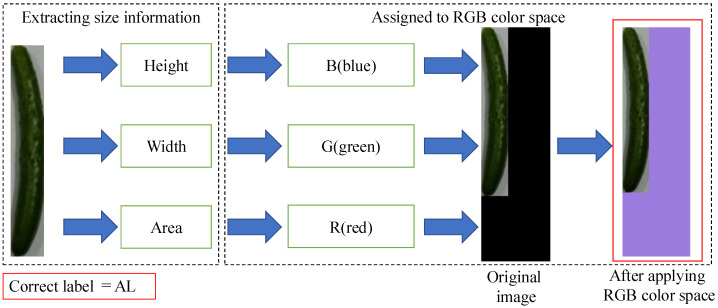
Overview of the method for generating training data using RGB color space.

We propose a method for generating training data that embeds numerical information about the height, width, and area of a cucumber indirectly within an image using RGB color space. Figure 8 illustrates an overview of the method for generating training data using RGB color space. The method with RGB color space calculates the height, width, and area of the cucumber using the same procedure as the method without RGB color space and pastes the cucumber image onto a 100 × 340 black background image.

**Figure 9:**
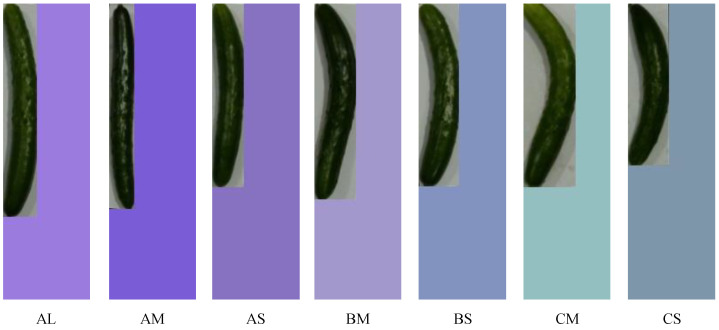
List of training data images after color transformation.

The RGB color space of the background image is then utilized to normalize these numerical values. The normalized values of height, width, and area are assigned to the B, G, and R channels, respectively. As a result, the background color of the image changes according to the length, curvature, and thickness of the cucumber. By embedding this information in the image, numerical values such as height, width, and area can be treated as part of the image information during the learning process. Finally, the method assigns a correct label to the transformed image in the same way as the first proposed method. Figure 9 shows a list of training data images after color transformation.

It can be observed that the background color varies with changes in cucumber length, curvature, and thickness, as well as differences in grades. The proposed method performs background color conversion during the data collection stage for training data used in model construction. Since color conversion is a relatively lightweight process, it is not expected to significantly increase the time required for creating training data.

### 2.7. Learning Model Generation

**Figure 10:**
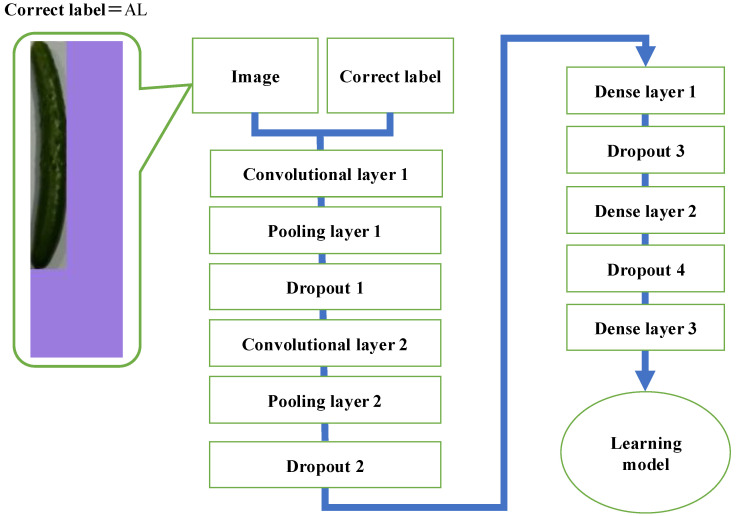
Flow of learning model creation in the method with RGB color space.

Figure 10 shows the flow of learning model creation in the method with RGB color space. First, images and correct labels created by the RGB color space-based training data generation method described in Section 2.6 are input to the feature extraction layer, which consists of four layers: two convolution layers and two pooling layers. At this stage, the input image is resized to 72 × 24. The learning model is then created by inputting the obtained features into a layer consisting of three fully connected layers. Four dropout layers [25,26] are added to suppress overfitting. Since the method with RGB color space embeds height, width, and area information into the background color of the image, the concatenated layers used in the method without RGB color space (shown in Figure 3) are not necessary.

Table 1 shows the Neural Network Parameters the method with RGB color space. Since model construction only involves loading images, the proposed method can achieve model creation in a time comparable to conventional methods.

#### 2.7.1. Dropout Layer

In this learning model, a dropout layer is used to suppress overlearning [27]. Dropout is a method to mitigate overlearning and improve the accuracy of learning by inactivating a certain percentage of nodes while learning using a neural network. In this paper, multiple patterns were tested to find the optimal drop rate in the dropout layer.

**Figure 11:**
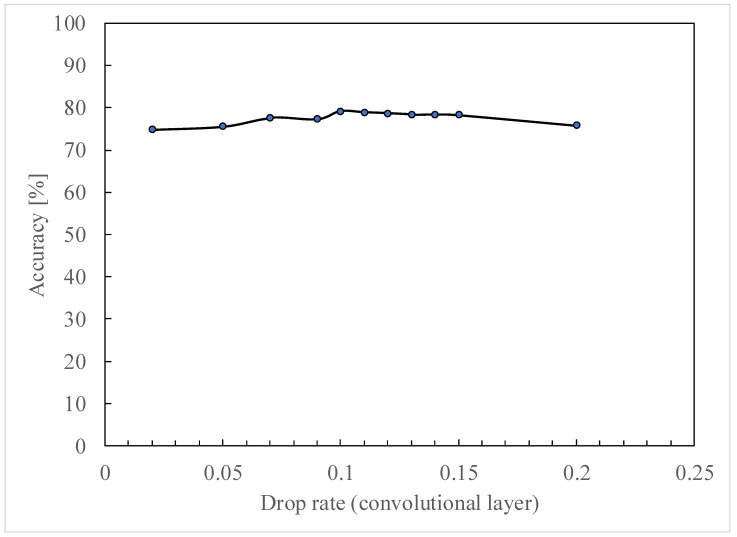
Results of the verification by varying the drop rate.

Figure 11 shows the results of the verification by varying the drop rate. The drop rate of the dense layer is fixed at 0.1 and the drop rate of the convolutional layer is varied, resulting in the maximum correct answer rate at 0.1. Subsequently, we observed that the percentage of correct answers decreased as the drop rate was increased. Therefore, a dropout layer with a drop rate of 0.1 is added to both the convolutional and dense layers as a combination of drop rates.

#### 2.7.2. Operation of Device

**Figure 12:**
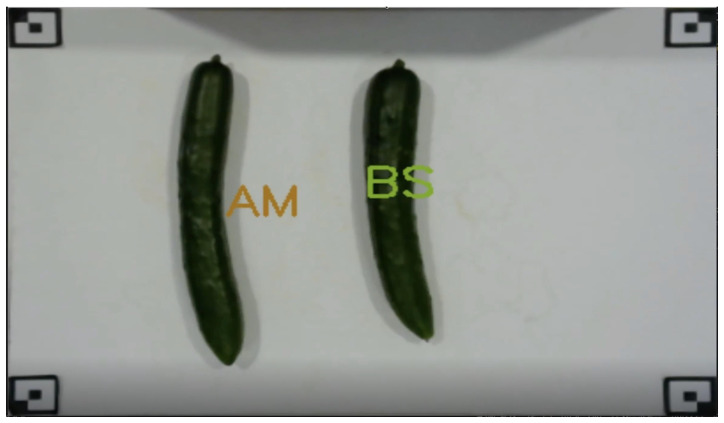
Classification screen on the device.

Figure 12 shows the classification screen on the device. Markers are placed on the four corners of the white board at intervals of 30 cm (length) and 40 cm (width). The cucumber is placed to avoid being covered by the four corner markers, and only the cucumber is clipped from the image captured by a webcam positioned directly above for classification. The distance between the camera and the cucumber at that time is not specifically determined. The reason is that the size information is obtained by the ratio of the distance between the markers and the size of the cucumber, due to the placement of the markers. After the grade classification is completed, the device informs the user of the correct grade by displaying the obtained grade name on the screen. By placing cucumbers between the markers, the device can also be used to determine the grade of multiple cucumbers. As for the intensity of the light in the room, the normal room brightness was assumed for the room after harvesting. No special light illumination was used. Table 2 shows the environment of the devices used for system creation.

## 3. Performance Evaluation

### 3.1. Evaluation Environment

We evaluated the judgment accuracy, recall, precision, and F-measure of the method without RGB color space and the method with RGB color space when the number of training cycles is used as a parameter. In this evaluation, the dropout rate, learning rate, and filter size were set to 0.1, 0.001, and 7 × 7, respectively. Additionally, the Adam optimizer was employed, and cross-entropy was used as the loss function. The number of training data images and the number of test data images used in the evaluation are shown in Table 3 and Table 4, respectively. The training and testing data in this study were created based on cucumbers harvested by farmers. Additionally, the farmers classified each cucumber’s grade based on their experience, and their classification results were used as the ground truth labels. Since the experiments were conducted using actual harvested cucumbers, the dataset is relatively small. However, we improved the reliability of our experiments by creating ten models.

**Figure 13:**
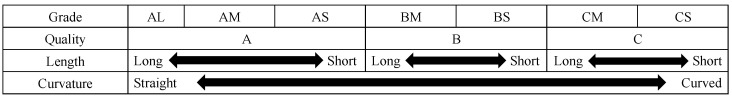
Types of grades used in the performance evaluation.

Each grade is based on an actual agricultural cooperative union, and there are seven grade types. Figure 13 shows the types of grades used in the performance evaluation. The degree of curvature of a cucumber determines whether it is classified as an A, B, or C cucumber. Straight cucumbers are classified as A, and as the degree of curvature increases, they are classified as B or C, in that order. Within each grade, such as Grade A, cucumbers are classified as L, M, or S, in descending order from the largest to the smallest.

The grade is determined by the combination of the degree of curvature and the size of these cucumbers. For example, the grade for straight and large cucumbers would be AL. The total number of images for each grade used in the study is 631, and the number of images for AL, AM, AS, BM, BS, CM, and CS are 84, 94, 93, 90, 92, 90, and 88, respectively. Because images of actually harvested cucumbers were used for the evaluation, the number of training data images for each grade varied. A total of 70 test images were used, with 10 images for each grade. The training data images were captured and created in a laboratory setting. The camera used was a Logitech web camera, C310 HD720P. The specifications of this camera are as follows: video resolution: HD720p, pixel count: 1.2 megapixels, maximum frame rate: 30 fps, and field of view: 60 degrees diagonal. Since no lighting corrections were applied, we believe the system can function adequately in general environments.

### 3.2. Evaluation Results

Table 5 shows accuracy, recall, precision, and F-measure for each method at various numbers of training cycles. The training data generation with RGB color space and the training data generation without RGB color space are w/ and w/o in Table 5, respectively. Ten learning models were created for each training cycle, and 70 test images were input into each learning model to measure accuracy, recall, precision, and F-measure.

It can be observed that the proposed method demonstrates high performance across all metrics, except when the training cycle is set to 10. These results indicate that the proposed method contributes to improving the performance of cucumber classification. It is considered that embedding size-related attributes in the RGB color space improves classification accuracy because it enables the processing of size information to be performed as image processing, which is a strength of CNNs. However, when the training cycle is 10, the performance of the proposed method declines compared to the method without RGB color space.

The reason for this is that when the number of training cycles is as low as 10, the proposed method suffers from underfitting and fails to extract sufficient features from the RGB color space. In contrast, the conventional method explicitly provides size information as numerical values, allowing it to directly capture size-related features. On the other hand, when the number of training cycles is more than 100, the proposed method can effectively extract features from images in which size-related information is embedded in the background color. This enables the generation of a high-quality learning model, which, as a result, likely contributed to improving the performance of cucumber classification.

We focus on accuracy, which is a crucial metric in cucumber classification. Looking at the highest-performing case, where the number of training cycles is 5000, the proposed method achieves an accuracy of 79.1%, while the method without RGB color space achieves 70.1%. This indicates that the proposed method attains 1.1 times the performance of the conventional method. Based on these results, it can be concluded that the proposed method is an effective approach for cucumber grading.

The performance evaluation was conducted based on the standards of a specific agricultural cooperative. This system can also be applied to other agricultural cooperatives. For example, when the agricultural cooperative changes, the grading criteria for cucumbers may change. However, this change only affects the relationship between cucumber images and their corresponding ground truth labels. Therefore, it is sufficient to create a grading model for each cooperative individually, making it easy to apply this system to other agricultural cooperatives.

## 4. Conclusions

This paper mainly proposed a method for embedding information related to cucumber length, bend, and thickness into the background space of cucumber images when creating training data. Specifically, this method encodes these attributes into the RGB color space, allowing the background color to vary based on the cucumber’s length, bend, and thickness.

Performance evaluation showed that the proposed method was high performance across all metrics such as accuracy, recall, precision, and F-measure, except when the training cycle is set to 10. This indicates that the proposed method is effective, provided that a sufficient number of training cycles is performed.

Accuracy is a crucial metric in cucumber classification. Focusing on the highest-performing scenario, where the number of training cycles is 5000, the proposed method achieves an accuracy of 79.1%, whereas the conventional method attains 70.1%. This corresponds to a 1.1-fold improvement in performance. These results demonstrate the effectiveness of the proposed method for cucumber classification.

As future work, we plan to explore a method where transfer learning is used to apply training data from one region to develop a classification model for a new region or agricultural cooperative association.

## Figures and Tables

**Table 1:** Neural Network Parameters.

Layer Type	Parameter	Value
Convolutional Layer 1	Number of Filters	8
	Filter Size	7×7
	Stride	[1, 1]
	Activation Function	ReLU
	Regularization	Batch Norm
Convolutional Layer 2	Number of Filters	16
	Filter Size	7×7
	Stride	[1, 1]
	Activation Function	ReLU
	Regularization	Batch Norm
Pooling Layer	Filter Size	2×2
	Type	Max Pooling
Dense Layer	Number of Units (Layer 1)	64
	Number of Units (Layer 2)	32
	Number of Units (Output Layer)	7
Other Parameters	Batch Size	100
	Max Steps	10,000
	Learning Rate	0.001
	Optimizer	Adam
	Loss Function	Cross Entropy

**Table 2:** The environment of the devices used for system creation.

OS	Windows10
Camera	Logicool C270n (Logitech, Tokyo, Japan)

**Table 3:** Number of training data images (production area N).

Total Number of Sheets: 631						
AL	AM	AS	BM	BS	CM	CS
84	94	93	90	92	90	88

**Table 4:** Number of test data images (production area N).

Total Number of Sheets: 70						
AL	AM	AS	BM	BS	CM	CS
10	10	10	10	10	10	10

**Table 5:** Accuracy, recall, precision, and F-measure of each method at various numbers of training cycles.

Numbers of Training Cycles	10		100		1000		5000		10,000	
Method	w/o	w/	w/o	w/	w/o	w/	w/o	w/	w/o	w/
Accuracy(%)	50	32.6	67.7	72.6	69.1	76.4	70.1	79.1	67.4	76.7
Recall(%)	50	32.6	67.7	72.6	69.1	76.4	70.1	79.1	67.4	76.7
Precision(%)	50.9	26.8	70.2	76.5	73	78.3	72.7	80.7	68.9	79.3
F-measure(%)	45.9	24.5	65.8	71.1	68.1	75.1	69.4	77.7	66.5	75.2

## Data Availability

Data are contained within the article.
